# Defining the therapeutic reference range for cariprazine

**DOI:** 10.1192/j.eurpsy.2023.1178

**Published:** 2023-07-19

**Authors:** M. Kapás, A. Horváth, D. Djuric, R. Csehi, Á. Barabássy

**Affiliations:** Gedeon Richter Plc., Budapest, Hungary

## Abstract

**Introduction:**

According to the Consensus Guideline, the “therapeutic reference range” (TRR) defines ranges of drug blood concentrations that specify a lower limit below which a drug-induced therapeutic response is unlikely to occur and an upper limit above which tolerability decreases or the therapeutic improvement ceases. The TRR can be obtained from concentration measurements (trough (pre-dose) plasma concentration under steady-state conditions) in studies at therapeutically effective doses.

**Objectives:**

The aim is to examine the TRR for cariprazine (CAR: 1.5 mg/day to 6 mg/day) in schizophrenia studies.

**Methods:**

The population based TRR for CAR is derived by PK/PD evaluation from phase 2/3 schizophrenia efficacy studies with sparse PK sampling. The population PK simulated TRR is compared to the actually measured values obtained from two PK studies. As the two active metabolites of cariprazine also contribute to the drug effect, plasma exposure is given for Total cariprazine (CAR + DCAR + DDCAR) and the parent drug (CAR).

**Results:**

PK/PD analyses demonstrated an increase in efficacy with increasing exposure. These efficacy results are related to Total cariprazine trough concentrations of ca. 30 nM and 100 nM that determine the lower and upper TRR limits. For the parent drug, the pre-dose mean plasma concentration at 6 mg/day was between 5.7-10 ng/mL in different studies, while at 1.5 mg/day it was 1.9 ng/mL.

**Conclusions:**

The TRR of the trough plasma levels at steady state is ca. 30 – 100 nM for Total cariprazine and ca. 2-10 ng/mL for the parent drug (unchanged drug) for schizophrenia treatment.

**Disclosure of Interest:**

M. Kapás Employee of: Gedeon Richter Plc., A. Horváth Employee of: Gedeon Richter Plc., D. Djuric Employee of: Gedeon Richter Plc., R. Csehi Employee of: Gedeon Richter Plc., Á. Barabássy Employee of: Gedeon Richter Plc.

